# Anodization Mechanism on SiC Nanoparticle Reinforced Al Matrix Composites Produced by Power Metallurgy

**DOI:** 10.3390/ma7128151

**Published:** 2014-12-19

**Authors:** Sonia C. Ferreira, Ana Conde, María A. Arenas, Luis A. Rocha, Alexandre Velhinho

**Affiliations:** 1 Materials Research Centre/Institute for Nanostructures, Nanomodelling and Nanofabrication (CENIMAT/I3N), Faculdade de Ciências e Tecnologia, FCT, Universidade Nova de Lisboa, 2829-516 Caparica, Portugal; E-Mails: sccf@campus.fct.unl.pt (S.C.F.); ajv@fct.unl.pt (A.V.); 2 Centro Nacional de Investigaciones Metalúrgicas (CENIM-CSIC), Avda. Gregorio del Amo, 8, 28040 Madrid, Spain; E-Mail: a.conde@cenim.csic.es; 3 Centre for Mechanics and Materials Technologies (CT2M), Research Group on Functionalized Materials and Surfaces Performance, Campus de Azurém, 4810-058 Guimarães, Portugal; 4 Departamento de Física, Faculdade de Ciências, Campus de Bauru, Universidade Estadual Paulista “Júlio de Mesquita Filho” (UNESP), Av. Eng. Luiz Edmundo Carrijo Coube, 14-01, 17033-360 Bauru, SP, Brazil; E-Mail: lrocha@fc.unesp.br; 5 Departamento de Ciência dos Materiais (DCM), Faculdade de Ciências e Tecnologia, Universidade Nova de Lisboa, Quinta da Torre, 2829-516 Caparica, Portugal

**Keywords:** aluminum matrix composite, powder metallurgy, anodizing, oxide film, nanosized reinforcements

## Abstract

Specimens of aluminum-based composites reinforced by silicon carbide nanoparticles (Al/SiC_np_) produced by powder metallurgy (PM) were anodized under voltage control in tartaric-sulfuric acid (TSA). In this work, the influence of the amount of SiC_np_ on the film growth during anodizing was investigated. The current density *versus* time response and the morphology of the porous alumina film formed at the composite surface are compared to those concerning a commercial aluminum alloy (AA1050) anodized under the same conditions. The processing method of the aluminum alloys influences the efficiency of the anodizing process, leading to a lower thicknesses for the unreinforced Al-PM alloy regarding the AA1050. The current density *versus* time response is strongly dependent on the amount of SiC_np_. The current peaks and the steady-state current density recorded at each voltage step increases with the SiC_np_ volume fraction due to the oxidation of the SiC_np_. The formation mechanism of the anodic film on Al/SiC_np_ composites is different from that occurring in AA1050, partly due the heterogeneous distribution of the reinforcement particles in the metallic matrix, but also to the entrapment of SiC_np_ in the anodic film.

## 1. Introduction

Hard ceramic particle-reinforced aluminum matrix composites (Al-MMCs) have received great interest due to their mechanical and physical properties; as a result, they are widely used to fabricate diverse components for the automotive and aerospace industries [1,2]. Al-MMCs can be reinforced with various oxides, carbides, nitrides and borides, such as SiC, Al_2_O_3_, B_4_C, TiC, TiB_2_, MgO, TiO_2_, AlN, BN and Si_3_N_4_ [3,4,5,6,7]. The literature is quite extensive on studying the system aluminum matrix reinforced with silicon carbide particles (Al/SiC_p_), in order to characterize its mechanical [8,9,10], wear [11,12], corrosion [13,14] and tribocorrosion [15,16] behavior, showing that the fraction volume and/or particle size of SiC_p_ play an important role in the composite behavior.

Powder metallurgy is a solid phase process for the production of particle-reinforced metal matrix composite (MMCs) [17]. Some advantages of solid phase powder metallurgy (PM) compared to casting, such as the improved uniform distribution of the reinforcement within the matrix, minimal solid-state reactions between the metal matrix and the ceramic reinforcement avoiding undesired phases and enhanced bonding between the constituents, have been reported [17,18,19].

The mechanical properties of the particle-reinforced MMCs are significantly affected by the amount and size of hard ceramic reinforcements [8,9,20]. The SiC nanometric particulate-reinforced Al-MMCs have shown an improvement in the mechanical properties when compared with reinforcement by SiC micrometric ceramic particles [20]. Reinforcement particle size has a strong effect on the failure mode of the MMCs. Large ceramic particles act as micro-concentrators of stress and give rise to cleavage in the particle, leading to failure of the composites [8]. Both tensile strength and ductility decrease with increasing particle size [8,9,20,21]. Similarly, agglomerates of particles degrade the mechanical properties [8,9,20,21,22], since they cannot transfer shear and tensile stress as a result of the lack of a metal matrix between the reinforcement particles forming the agglomerate [22].

In addition to the mechanical properties, the type of particle-reinforcement, their size and their amount have also a strong influence on Al-MMCs’ corrosion resistance [23,24,25,26]. The different electrochemical properties between the metallic matrix and the ceramic reinforcement make MMCs more susceptible to corrosion, and therefore, additional protection is required.

Anodizing is one of the most widely-used surface protective treatments for aluminum alloys [27]. Sulfuric acid anodizing has been widely studied by several authors for the protection of Al/SiC-MMC with uneven effectiveness depending on the alloy [23,24,25,28,29]. Other anodizing electrolytes have been also used for Al-MMC, showing that Al/AlN-MMC anodized in alkaline solutions presents better results than hard sulfuric anodized SiC/Al-MMC [29]. However, the literature is scarce on the fabrication of anodic layers on nanoparticle-reinforced Al-MMC using environmentally-friendly procedures, such as tartaric-sulfuric acid (TSA), recently adopted by the European aircraft industry [30,31,32,33].

In general, the poor corrosion resistance of the anodic films fabricated on Al/MMC is due to their discontinuity and non-uniform thickness [24,28,29]. He *et al.* [34] studied the effect of the size of reinforcement particles on the thickness of the anodic layers on Al(2024)/SiC-MMC, showing that the anodic layers are thicker for bigger particles, with an average diameter of 10 µm, but also, they have a lack of uniformity in thickness more remarkable than those fabricated on MMC with SiC_p_ of a smaller diameter, 3.5 µm.

Unlike the mechanical properties, where the use of finer reinforcement particles has demonstrated to enhance the performance of the MMC [8,9,20,21,22], the influence of the reinforcement nanoparticles on the anodizing process of MMC has been scarcely studied.

In the present work, the anodizing process in tartaric-sulfuric acid (TSA) electrolyte has been used to grow anodic oxide layers on Al-MMC reinforced with SiC nanoparticles produced by PM. The influence of different volume fractions of nanosized SiC reinforcement particles on the morphology, composition and thickness of the anodic film grown on Al/SiC_np_-MMCs substrates is discussed.

## 2. Results and Discussion

### 2.1. Raw Materials Characterization

**Figure 1:**
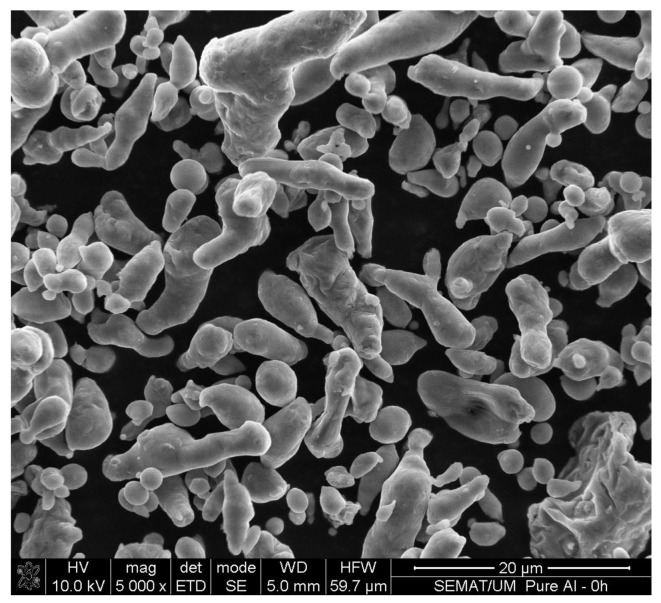
Morphology of as-received Al powders.

Figure 1 shows the morphology of the as-received Al powders, which exhibit a mixture of cylindrical and spherical shapes. Table 1 gathers the particle size distribution determined by laser diffraction (low angle laser light scattering (LALLS)); the average particle size was 7.70 ± 5.57 µm.

**Figure 2:**
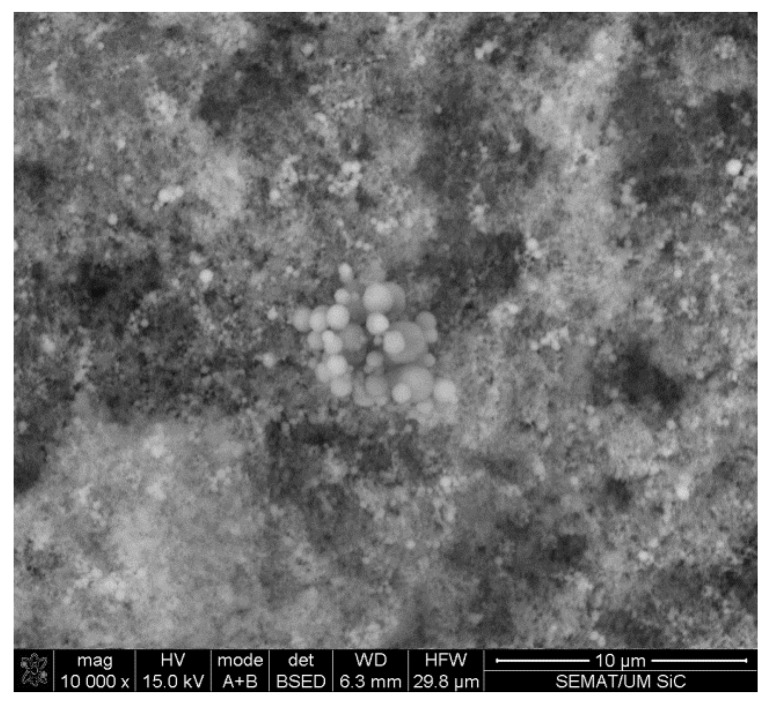
Morphology for the as-received SiC powders.

Figure 2 shows the morphologies of the as-received SiC powders. The particles are spherical, and the sizes varied from a few microns to tenths of nanometers. LALLS results show a multimodal particle size distribution: a set of fine particles of 0.07-µm average size and two sets of larger particles (average sizes 0.50 and 2.40 µm, respectively, as individual size or as agglomerates), as shown in Table 1.

Table 2 shows the different composition of the powders after the milling determined by XRF. After 1 h of milling, the unreinforced Al powder shows increased Si and Fe contents [7]. As was expected, when increasing contents of SiC powder were mixed into the Al powder, the overall Si content increased. Moreover, there is also an increase in Fe and Mn due to contamination by the steel balls used during the ball-milling.

**Table 1:** Particle size distribution of as-received Al and SiC powders.

Powders	Average particles size distribution		
As-Received Al	7.70 ± 5.57 µm		
As-Received SiC	0.07 µm	0.50 µm	2.40 µm

**Table 2:** XRF results of the composition of mixture powders (wt%).

Material		Al	Si	Fe	Cu	Mn	Cr
As-received Al		99.60	0.13	0.18	0.01	>0.01	>0.01
60-min milled	Al	99.50	0.20	0.21	0.01	>0.01	>0.01
	Al/1 vol% SiC_np_	98.00	1.69	0.20	0.01	0.03	>0.01
	Al/5 vol% SiC_np_	94.50	5.05	0.26	0.03	0.13	>0.01
	Al/10 vol% SiC_np_	89.80	9.66	0.31	0.02	0.14	0.02

### 2.2. Microstructure of Al/SiC_np_ Composites after Sintering

**Figure 3:**
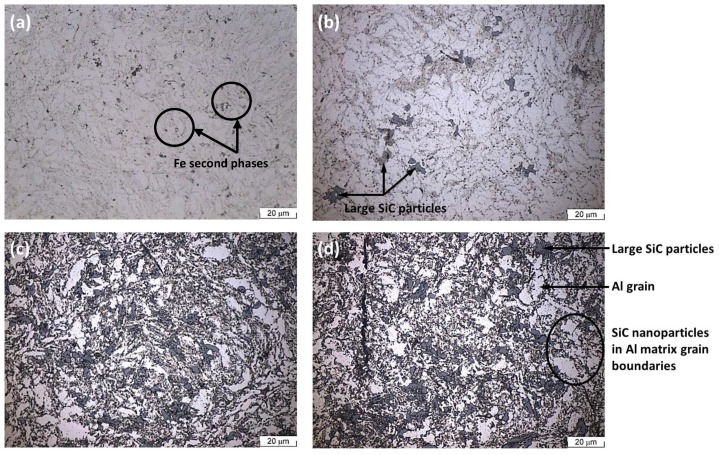
Optical micrographs: (**a**) Unreinforced Al-powder metallurgy (PM) alloy; (**b**) Al/1 vol% SiC_np_; (**c**) Al/5 vol% SiC_np_; (**d**) Al/10 vol% SiC_np_. All of the specimens were etched in a solution of 0.5 vol% hydrofluoric acid (HF) for 30 s.

The optical micrographs in Figure 3 show the morphology of the unreinforced Al-PM alloy and Al/SiC_np_ composites etched by 0.5 vol% hydrofluoric acid (HF). The microstructure of the unreinforced Al-PM alloy (Figure 3a) shows the presence of second phases, enriched in Fe from the milled Al powders. The Al/1 vol% SiC_np_ composite (Figure 3b) shows the presence of large silicon carbide particles. The microstructure of the Al/5 vol% SiC_np_ and Al/10 vol% SiC_np_ composite was very similar (Figure 3c,d). Both composites show the typical microstructure of a PM processed material. As can be seen, the reinforcement particulates are distributed in the aluminum matrix, and due to a significant difference between the particle size of the SiC powder and the Al powder (Figure 1 and Figure 2), the SiC nanometric particles tend to fill in the gaps between aluminum powders during powder mixing. Some particles aggregate to form clusters in the Al matrix. This effect is most evident in the composite with higher SiC_np_ contents (5 and 10 vol%).

**Figure 4:**
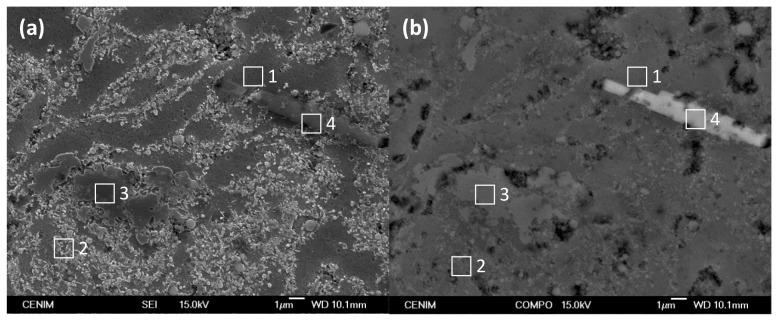
(**a**) SEI image showing the surface appearance of the Al/5 vol% SiC_np_ composite etched in 0.5 vol% HF for 30 s; (**b**) BEI image of the same area. Elemental analysis of the phase constituents is given in Table 3.

Figure 4 shows the constituent phases of the microstructure of the Al/5 vol% SiC_np_ in secondary electron imaging (SEI) and in backscattered electron imaging (BEI) mode, respectively. The corresponding chemical compositions are listed in Table 3. The dark grey areas observed in SEI mode (identified by No. 1 in Figure 4a) correspond to the aluminum matrix showing the lowest silicon content. In the same figure, light grey areas (e.g., No. 2) correspond to zones densely reinforced with small SiC nanoparticles. The larger particles, more clearly observed in BEI mode, (No. 3 in Figure 4b), are identified as SiC. Finally, in all of the specimens produced, including the unreinforced Al-PM alloy, the elongated white phases, (No. 4 in Figure 4b), are Fe-rich precipitates resulting from the presence of Fe impurities in the aluminum powder and the erosion of the grinding balls during the fabrication process. These precipitates are Al/Fe intermetallics, the size of which depends on the sintering temperatures [35].

**Table 3:** EDX results at different locations shown in Figure 4 (at%).

at%	C	O	Al	Si	Mn	Fe
Spectrum 1	2.49	3.22	92.64	1.76	–	–
Spectrum 2	20.12	10.33	52.02	17.53	–	–
Spectrum 3	8.73	7.40	32.90	50.97	–	–
Spectrum 4	8.09	13.97	56.29	12.14	0.85	8.65

### 2.3. Surface Modification of Al/SiC_np_ by Alkaline Etching and De-Smutting

**Figure 5:**
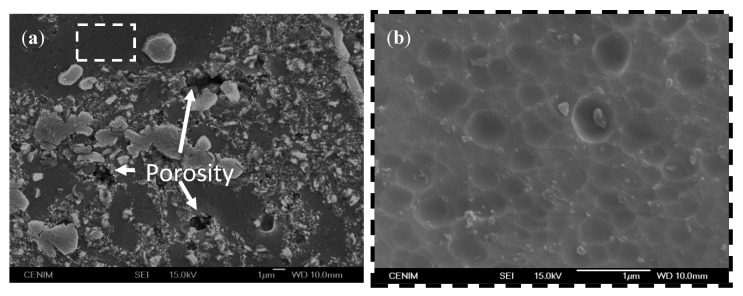
(**a**) SEM image of the Al/5 vol% SiC_np_ after alkaline etching and de-smutting; (**b**) close view of the boxed area.

Before the anodizing process, the specimens were pre-treated in an alkaline bath in sodium hydroxide solution and then de-smutted in nitric acid solution. This is a common pre-treatment used in the aircraft industry for preparing the aluminum alloys surfaces before the anodization. The alkaline treatment promotes the formation of a smut layer composed of oxides, which is subsequently removed by immersion in the acid solution de-smutting. Figure 5a shows the pre-treated surface topography of the Al/5 vol% SiC_np_ composite. After the pretreatment, the surface reveals a high concentration of SiC particles, as well as some porosity in those areas where particles are concentrated. At higher magnifications, the areas with a lower presence of reinforcement particles (dotted line rectangle of Figure 5a) show a grooved surface, pointing out the composite dissolution during the alkaline etching; Figure 5b. This area is not entirely free of particles, since finer reinforcement particles are distinguished on the surface.

These pre-treated surfaces will be subsequently anodized in TSA.

### 2.4. Anodizing of Al/SiC_np_ Composites in TSA Bath

**Figure 6:**
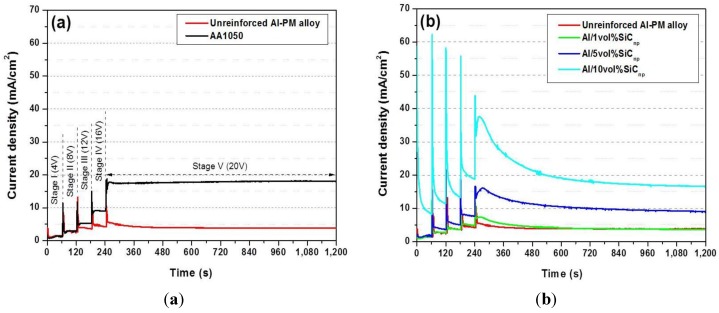
Comparison of the anodizing current-time responses at 20 V in a TSA bath at 35 °C: (**a**) unreinforced Al-PM alloy and commercial AA1050; (**b**) Al/SiC_np_ composites prepared by PM.

During the anodizing process performed in TSA under controlled voltage conditions, the current density-time response is recorded for a better control of the process. Figure 6a shows the current transients corresponding to unreinforced Al-PM alloy and the commercial AA1050 aluminum alloy. The current density-time responses corresponding to the Al/SiC_np_ composites are shown in Figure 6b.

All of the current density curves show five stages corresponding to each 4-V step applied. At the beginning of each stage, there is a surge in current, followed by decay towards a steady value. This surge arises from rapid thickening of the anodic oxide in response to the increase of field following the voltage step [36]. Additionally, in the specimens containing SiC_np_, a peak is recorded in Stage V at 270 s, approximately, just before the steady state at 20 V is reached.

**Figure 7:**
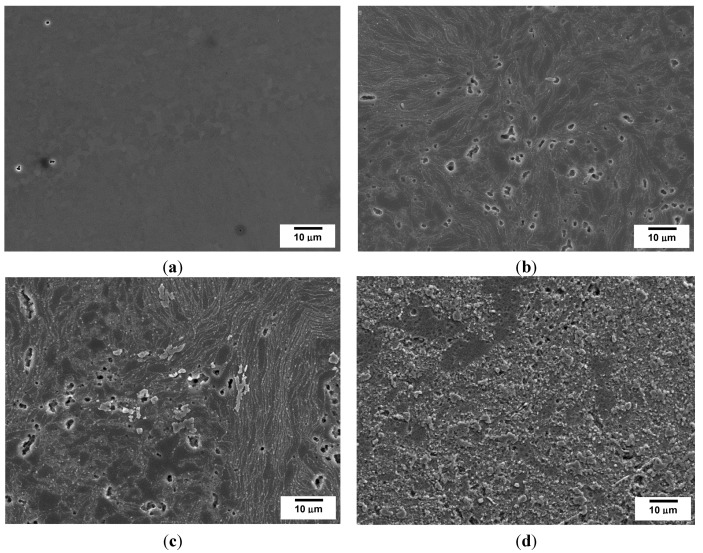

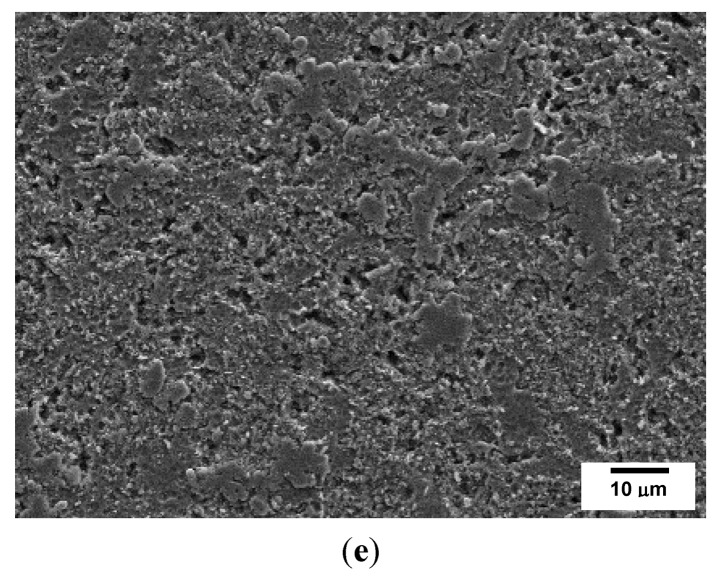
SEM micrographs after alkaline etching and de-smutting for: (**a**) commercial AA1050; (**b**) unreinforced Al-PM alloy; (**c**) Al/1 vol% SiC_np_; (**d**) Al/5 vol% SiC_np_; (**e**) Al/10 vol% SiC_np_.

The comparison of the current density-time response corresponding to the unreinforced Al-PM and the commercial AA1050 aluminum alloy reveals significant differences; Figure 6a. The current density achieved at steady state is around 18 mA·cm^−2^ for AA1050 and 4 mA·cm^−2^ for unreinforced Al-PM alloy. This result suggests that even though their chemical compositions are similar, the processing method of the aluminum alloys has a remarkably influence on the anodizing process. The residual porosity of the specimens obtained by PM might influence the current density-time responses (see Figure 7). The residual porosity significantly increases the effective area available during the anodizing process, but it also modifies the electrical conductivity of the material.

In fact, the conductivity seems to be the key parameter to explain the different behavior observed between the AA1050 and unreinforced Al-PM alloy. In the commercially pure aluminum alloy, the efficiency of the anodizing process is the highest, since all of the applied charge is mostly used to grow the alumina film. In this sense, the lower current density recorded for the unreinforced Al-PM alloy indicates a lower efficiency of the anodizing process regarding the commercial alloy.

The current density-time response, Figure 6b, recorded for the unreinforced Al-PM alloy and Al/1 vol% SiC_np_ have similar features in the five stages. Surges, decays and plateaus of current density are analogous, presenting in Stage V a steady current density of about 4 mA·cm^−2^.

With the increase of the reinforcement volume fraction of SiC_np_ in Al composites, the current densities of each stage increase, and the steady state is more difficult to achieve. Moreover, in the Al/10 vol% SiC_np_, the curve shows that the current density exhibits a continuous decay until the next voltage step is applied, and the steady state is only reached in the last voltage step (Stage V). The Al/SiC_np_ composites reinforced with 5 and 10 vol% achieve a steady current density of about 9 and 16.5 mA·cm^−2^, respectively, at 1200 s. At the beginning of Stage V, after the initial current time surge, the composites reinforced with 5 and 10 vol% clearly reveal a peak that decreases slowly until reaching the steady state. This peak appears related to the presence of higher SiC_np_ content, suggesting that the higher the content of reinforcement particles, the higher the contribution of the SiC_np_, which is being oxidized at the applied voltage. Therefore, the changes observed on the current density *vs.* time responses suggest that the incorporation of the nanometer SiC_p_ in the alloy varies the formation mechanism and growth rate of the anodic layer regarding the unreinforced Al-PM alloy.

It has to be mentioned that the residual porosity observed on the specimens with 5 and 10 vol% after pre-treatment (Figure 7d,e) also affects the electrical conductivity of these materials and, therefore, the anodizing process. Moreover, Srivastava *et al.* [36] and Padmavathi *et al.* [37] found that the electrical conductivity of Al/SiC_p_ decreases with the increase in the volume fraction of the SiC_p_, not only due to the addition of a phase with lower conductivity than the Al matrix, but also because when high volume fractions of reinforcement particles are present, reinforcement clusters and porosity are often observed to decrease the electrical conductivity of the composite material.

**Figure 8:**
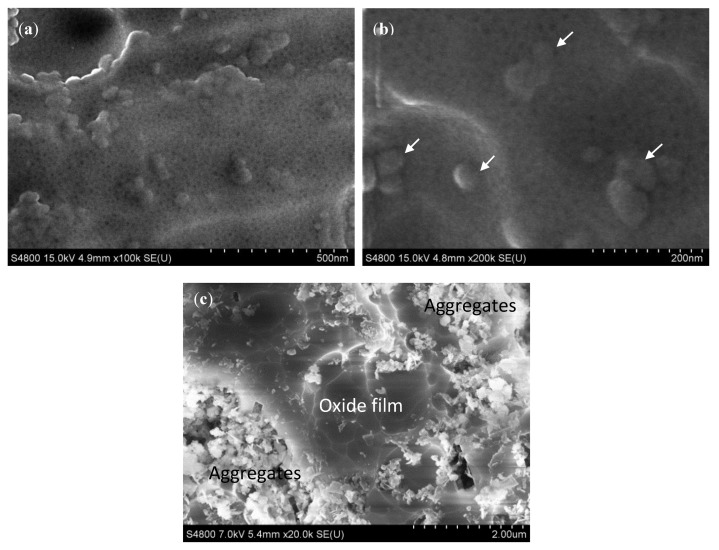
Top-view of the anodic film formed on: (**a**,**b**) Al/5 vol% SiC_np_; (**c**) Al/10 vol% SiC_np_.

Figure 8a shows the top view of the porous anodic film grown on the Al/5 vol% SiC_np_. The average pore diameter ranges between 5 and 15 nm. These values are similar in all of the specimens evaluated in this work. Thus, the pore diameter is not influenced by the reinforcement volume fraction or the processing method of the aluminum alloys. These values are in the same range as those reported in the literature for some aluminum alloys anodized under similar conditions [38,39]. Moreover, the surface of the 5 vol% SiC_np_ specimen also shows rounded morphologies of 30–80 nm in diameter that appear to be the SiC particles embedded in the film, as can be observed in Figure 8b (white arrows).

Conversely, when the reinforcement volume fraction increases up to 10 vol% SiC_np_ the top view of the anodized surface shows great areas of particle aggregates. In these regions, the film is not formed; close to them, there are other areas covered by the anodic film; Figure 8c. Thus, the highest amount of reinforcements in these composites hinders the formation and growth of the film.

**Figure 9:**
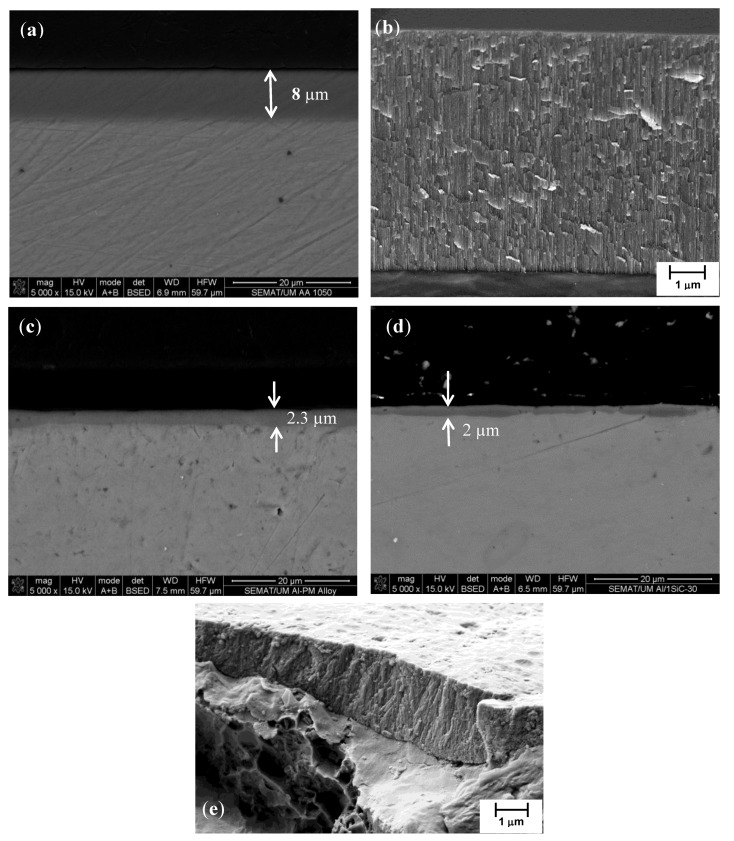
SEM micrographs of cross-sections of the anodic films formed on: (**a**,**b**) AA1050; (**c**) unreinforced Al-PM alloy; (**d**,**e**) Al/1 vol% SiC_np_.

The cross-sections of the anodic layer grown on AA1050, unreinforced Al-PM alloy and Al/1 vol% SiC_np_ composite are shown in Figure 9. On the AA1050 substrate, a relatively uniform oxide film of 8 µm, approximately, was formed (Figure 9a,b), Additionally in Figure 9b,e the characteristic porous structure growing perpendicularly to the substrate is observed for the oxide layer grown on AA1050 and on 1 vol% SiC_np_.

Conversely, for unreinforced Al-PM alloy (Figure 9c) and for Al/1 vol% SiC_np_ (Figure 8d,e), the oxide films are thinner and have a non-uniform thickness. The thickness of the anodic film is about 2.0–3.0 µm for the unreinforced Al-PM alloy and 0.6–2.1 µm for Al/1 vol% SiC_np_.

**Figure 10:**
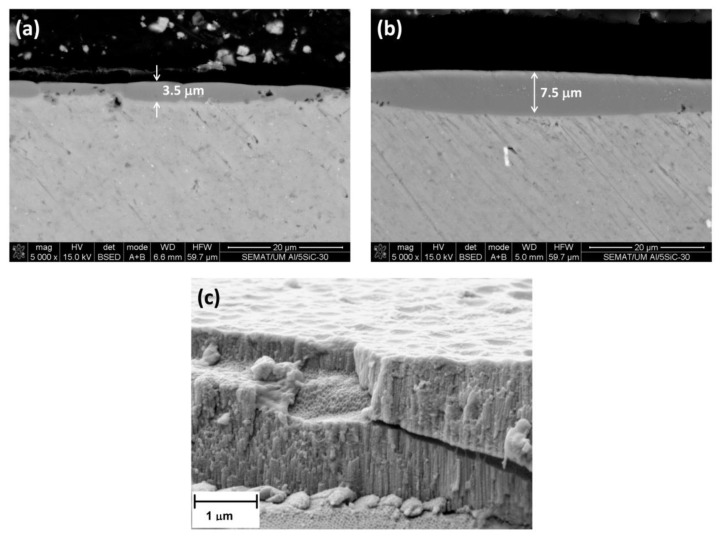
Cross-sections of the anodic films formed on Al/5 vol% SiC_np_ specimens: (**a**,**b**) areas with a non-uniform thickness film; (**c**) microstructure of the porous anodic layer.

For Al/5 vol% SiC_np_, the anodized specimen is much more heterogeneous. The thickness of the anodic film varies from 0.5 to 7.5 µm, depending on the area (Figure 10a,b). This large variation of film thicknesses is due to the heterogeneous distribution of SiC_np_ in the Al matrix, as was observed in Figure 5a,b). Figure 10a shows dark regions within the cross-section, the size of which is much higher than the SiC_np_. This suggests that, in fact, these morphologies appear to be cavities associated with the partially oxidized SiC nanoparticles/clusters. Several authors [25,40] have demonstrated that the anodic oxidation of silicon particles proceeded at a significantly reduced rate compared with that of the adjacent aluminum matrix. The occlusion of the partly oxidized particle in the film appears to be associated with cavities above the silicon particles, which had sizes and shapes dependent on the particle morphology. The silicon particles oxidize as a result of the O^2−^ ingress under the high field, and the growth of the anodic SiO_2_ rim around the particle proceeds with oxygen generation, presumably as a result of the semi-conducting nature of the Si-O bond. As a consequence, oxygen gas-filled voids develop above the oxidizing particles. In areas remote from SiC particles, regular porous anodic film develops.

Figure 10c shows the cross-section of the anodic film grown on the Al/5 vol% SiC_np_ where the characteristic pores perpendicular to the substrate are clearly seen on the anodized specimen.

In the anodizing process, either the amounts of particles or their sizes are important parameters. Smaller/nanoscale reinforcing particles hinder the film formation, but do not interrupt the continuity of the film. Thus, a more uniform film thickness can be achieved when nanosized SiC particles are uniformly distributed throughout the Al matrix, rather than microsized particles [25]. However, the film growth is restricted where there is a high content of SiC_np_ or these are heterogeneously distributed, forming agglomerates, since these clusters of particles shield the matrix.

This work has shown that Al composites reinforced with SiC nanoparticles produced by PM can be anodized, resulting in the growth of a continuous and potentially protective oxide film. Nevertheless, the amount of reinforcing SiC_np_ should be limited to 5 vol%, avoiding the formation of aggregates in the Al matrix, since there is no film in the areas with high concentration/aggregates of SiC_np_.

## 3. Experimental Section

### 3.1. Materials

Particle size distributions of raw materials, *i.e.*, as-received pure Al and as-received SiC powders, supplied by Goodfellow (Cambridge, UK), were measured by LALLS method using a Coulter LS 230 (Coulter Electronics, Krefeld, Germany) equipped with the PIDS technique (polarization intensity differential scattering).

The Al/SiC_np_ composites were produced by a powder metallurgy method and were prepared with different reinforcement volume fractions, namely 1, 5 and 10 vol%. For comparison purposes, an unreinforced Al-PM alloy specimen was also produced. Proper proportions of the powders were placed in a high-energy ball mill for 60 min, together with 5 mm diameter AISI (American Iron Steel Institute) 52100 steel balls. The total powder mass was 11 g, the ball to powder weight ratio being 2:1. Acetone was used as a process control agent (0.05 mL·g^−1^). The milled powders were consolidated by cold pressing at 200 MPa for 1 min into bulk specimens of 30 mm in diameter.

The chemical composition of the green compact specimens was measured using a Philips Analytical sequential X-ray fluorescence (XRF) spectrometer Model X’Unique II (Philips, Amsterdam, The Netherlands). In the sintering step, the green compact specimens are placed on a high-vacuum atmosphere furnace at 650 °C for 1 h, with a heating rate of 4 °C min^−1^.

### 3.2. Metallographic Preparation and SEM Analysis

The sintered specimens were ground to 1200 grit emery paper and polished to a 1-µm diamond suspension. In order to identify any second phases present, the specimens were etched by 0.5 vol% hydrofluoric acid (HF) for 30 s.

The morphology of the specimens was investigated using an optical microscope (OM) and scanning electron microscopes with energy dispersive X-ray (EDX): a JEOL 6500F (Musashino 3-chomeAkishima, Tokyo, Japan), a Hitachi S-4800 (Hitachi, Chiyoda-Ku, Tokyo, Japan), a NanoSEM-FEI Nova 200 (FEG/SEM, Nova, Hillsboro, OR, USA) and a ZEISS Auriga CrossBeam workstation (Carl Zeiss Microscopy GmbH, Jena, Germany).

### 3.3. Pretreatment and Anodizing Surface Treatment

The pretreatment process of the specimen prior to anodizing comprised the following steps: degreasing in ethanol, alkaline etching for 30 s in 0.5 M NaOH solution at 40 °C and de-smutting for 15 s in a 7.2 M nitric acid solution at room temperature. After each step, the specimens were gently rinsed with distilled water and dried.

The specimens were anodized in a two-electrode cell in 0.4 M H_2_SO_4_/0.5 M tartaric acid solution at 20-V stepped-voltages and at a temperature controlled at 35 °C. Consecutive voltage steps of 4 V for 60 s were applied up to a final value of 20 V, which was then maintained constant for 1200 s until completion of the anodizing process. A platinum mesh was used as a cathode. After anodizing, the specimens were immediately immersed for 10 min in deionized water at room temperature to remove any acid within the porous layer, then finally dried.

For comparison purposes, a commercial electropolished AA1050 sheet (minimum of 99.5% Al, with Fe < 0.30% and Si < 0.20% as principal alloying elements) was pre-treated and anodized under the same conditions as Al/SiC_np_ composites.

## 4. Conclusions

- Specimens of unreinforced Al-PM alloy and nanosized Al/SiC_p_ composites produced by PM were anodized in tartaric-sulfuric acid at 20 V to study the ability of this surface treatment to fabricate homogeneous anodic films with potential protective properties for the SiC_np_-reinforced Al-MMCs.
- The current density *versus* time response reveals that the SiC_np_ reinforcement volume fraction influences the anodizing mechanism.
- The increasing content of nanosized SiC particles in Al composites induces a rise of the current density values due to the partial oxidation of the SiC_np_. These particles are finally occluded in the film due to the preferential oxidation of the surrounding Al matrix. The presence of gas-filled voids throughout the cross-section of the anodizing film reveals the uniform entrapment of the SiC_np_ within the anodic film.
- SiC particles in the nanoscale range and with contents of about 1% and 5% decrease the efficiency of the anodizing process and hinder the film formation, but still, a continuous anodic oxide layer covers the surface; conversely, if the content is too high (10%), particles agglomerate, shielding the matrix, and the film is not formed.
